# Novel endoscopic suture anchor device for scarred perforation closure: a survival porcine pilot study

**DOI:** 10.1055/a-2723-1780

**Published:** 2025-11-14

**Authors:** Jiancong Feng, Yaqi Zhai, Zhenyu Liu, Enqiang Linghu

**Affiliations:** 1 651943 Department of Gastroenterology, The First Medical Center of Chinese PLA General Hospital, Beijing, China; 2 Department of Gastroenterology, 96605 Military Hospital, Tonghua, China

Delayed perforation after endoscopic submucosal dissection (ESD), although rare, is a serious complication whose management is complicated by inflammation and scarring 1 . The efficacy of conventional through-the-scope clips (TTSCs) is limited by poor grasping forces and spontaneous dislodgement, resulting in suboptimal outcomes, especially in necrotic or inflamed tissue 2 3 . The novel endoscopic suture anchor device, designed on the TTSC platform with rotational tissue-penetrating capability, has demonstrated feasibility for closing ESD defects 4 5 . This study aims to assess the device's feasibility for closing scarred perforation in a porcine model.

A 2.5-cm ESD defect was created on the gastric body in an in vivo porcine model, and endoscopy at week 4 showed scar formation at the wound site ( Fig. 1 ). A perforation was intentionally created at the scar site, but TTSC closure attempts failed due to clip slippage, reflecting clinical challenges. The preloaded suture anchor was advanced through the endoscopic channel. Rotation of the handle screwed the anchor into the scar tissue, and pressing the handle deployed it, establishing a purse-string configuration for perforation closure ( Fig. 2 , Video 1 ). During the procedure, the fifth and sixth suture anchors became dislodged from the scar tissue. Final closure was successfully achieved with seven anchors.

**Fig. 1:**
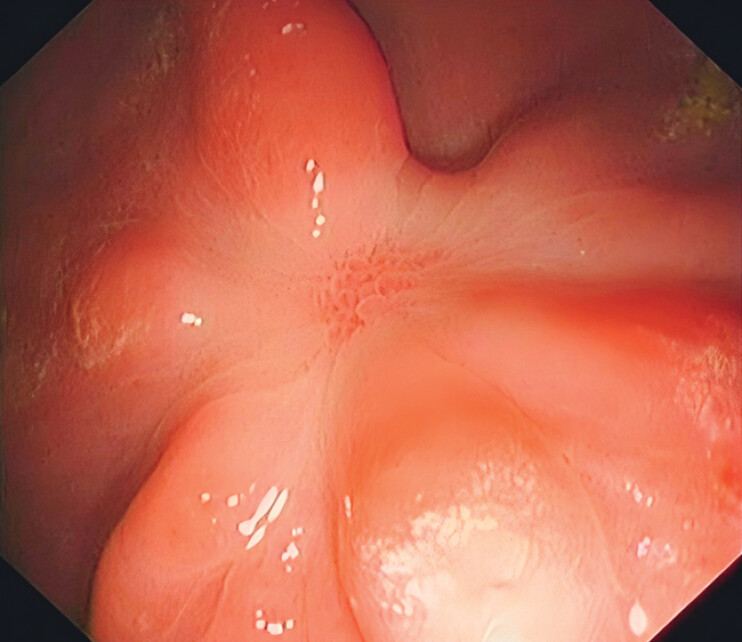
Endoscopic view showing scar formation.

**Fig. 2:**
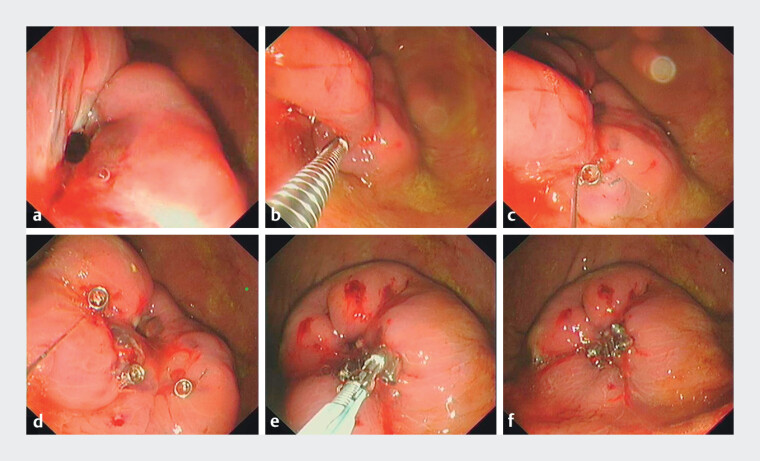
Endoscopic closure of a scarred perforation using novel suture anchors. **a** A scarred perforation. **b** Rotational insertion of the suture-loaded anchor into fibrotic tissue. **c** Implanted suture anchor position within the fibrotic tissue. **d** Suture anchors deployed in the purse-string configuration. **e** Cinching device advancement along suture. **f** Complete perforation closure achieved.

Endoscopic suture anchor closure of a scarred perforation in a porcine model.Video 1

Postoperative 1-week complete blood count and repeat endoscopy confirmed the absence of delayed bleeding or perforation. At 4 weeks, endoscopic examination demonstrated partial suture anchor retention with satisfactory healing at the wound site ( Fig. 3 ). The porcine model was euthanized for tissue harvesting. Hematoxylin eosin staining revealed collagenous connective tissue proliferation at the repair site ( Fig. 4 ).

**Fig. 3:**
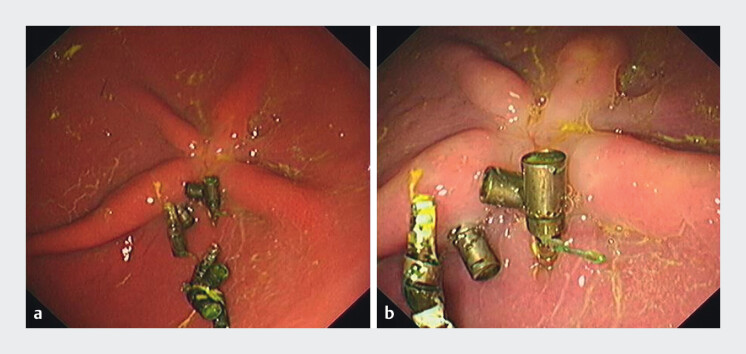
Endoscopic follow-up at 4 weeks postoperatively. **a** Partial suture anchors retained in situ. **b** Satisfactory healing at the wound site.

**Fig. 4:**
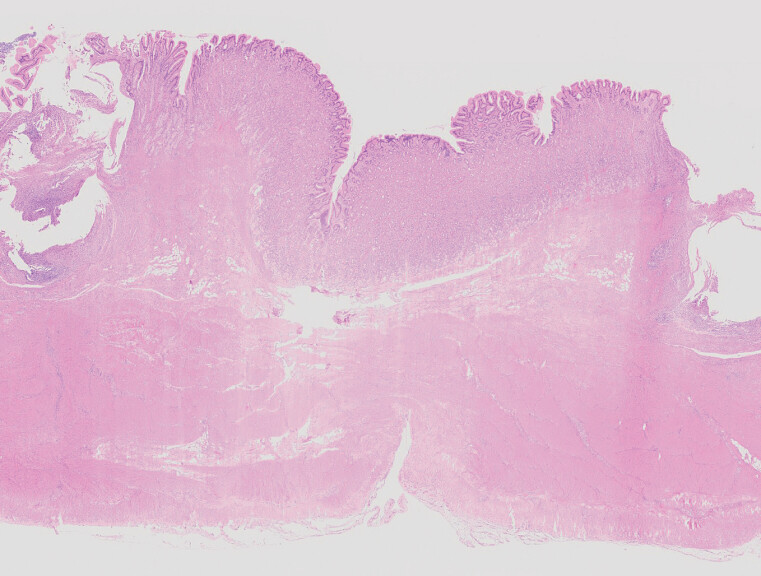
HE staining: Collagen deposition at the repair site. HE, hematoxylin eosin.

In the cases of premature release or dislodgement of suture anchors, additional anchors can be placed along the suture to complete the closure. This study demonstrates the feasibility of utilizing the device for closing scarred perforations; further studies are required.

Endoscopy_UCTN_Code_TTT_1AO_2AO

## Notes

Endoscopy E-Videos https://eref.thieme.de/e-videos
*E-Videos* is an open access online section of the journal *Endoscopy* , reporting on interesting cases and new techniques in gastroenterological endoscopy. All papers include a high-quality video and are published with a Creative Commons CC-BY license. Endoscopy E-Videos qualify for HINARI discounts and waivers and eligibility is automatically checked during the submission process. We grant 100% waivers to articles whose corresponding authors are based in Group A countries and 50% waivers to those who are based in Group B countries as classified by Research4Life (see: https://www.research4life.org/access/eligibility/ ). This section has its own submission website at https://mc.manuscriptcentral.com/e-videos .
